# Genetic polymorphisms linked to susceptibility to malaria

**DOI:** 10.1186/1475-2875-10-271

**Published:** 2011-09-19

**Authors:** Adel Driss, Jacqueline M Hibbert, Nana O Wilson, Shareen A Iqbal, Thomas V Adamkiewicz, Jonathan K Stiles

**Affiliations:** 1 Department of Microbiology, Biochemistry and Immunology, Morehouse School of Medicine, Atlanta, Georgia, USA; 2 Department of Urology, Winship Cancer institute, Emory University, Atlanta, Georgia, USA; 3 Department of Family Medicine, Morehouse School of Medicine, Atlanta, Georgia, USA

## Abstract

The influence of host genetics on susceptibility to *Plasmodium falciparum *malaria has been extensively studied over the past twenty years. It is now clear that malaria parasites have imposed strong selective forces on the human genome in endemic regions. Different genes have been identified that are associated with different malaria related phenotypes. Factors that promote severity of malaria include parasitaemia, parasite induced inflammation, anaemia and sequestration of parasitized erythrocytes in brain microvasculature.

Recent advances in human genome research technologies such as genome-wide association studies (GWAS) and fine genotyping tools have enabled the discovery of several genetic polymorphisms and biomarkers that warrant further study in host-parasite interactions. This review describes and discusses human gene polymorphisms identified thus far that have been shown to be associated with susceptibility or resistance to *P. falciparum *malaria. Although some polymorphisms play significant roles in susceptibility to malaria, several findings are inconclusive and contradictory and must be considered with caution. The discovery of genetic markers associated with different malaria phenotypes will help elucidate the pathophysiology of malaria and enable development of interventions or cures. Diversity in human populations as well as environmental effects can influence the clinical heterogeneity of malaria, thus warranting further investigations with a goal of developing new interventions, therapies and better management against malaria.

## Introduction: severity of malaria is influenced by host genetics

*Plasmodium falciparum *malaria is a major cause of mortality and morbidity, particularly in endemic areas of sub-Saharan Africa [1]. The disease aetiology is variable and is attributable to environmental factors, host genetics and parasite virulence [2]. Variations in severity of *P. falciparum *infections considered as different phenotypes include hyper or asymptomatic parasitaemia (proportion of red blood cells that are parasitized), severe malaria anaemia (SMA) and cerebral malaria (CM). Host genetic factors contribute to the variability of malaria phenotypes [3] and thus, should help to determine some of the mechanisms involved in susceptibility to *P. falciparum *infection. The knowledge gained since 1980s using molecular genetics approaches has produced undisputed evidence about polymorphisms associated with malaria resistance and their complex interactions. Indeed, several gene mutations and polymorphisms in the human hosts confer survival advantage and have increased in frequency through natural selection over generations. These include sickle cell trait (HbAS) and haemoglobinopathies such as thalassaemias and glucose-6-phosphate dehydrogenase (G6PD) deficiency (Table 1) [4]. In the last decade, the development of molecular biology technologies and the completion of the human genome project have identified other loci that appear to directly or indirectly affect malaria susceptibility by modulation of the immune response, or by interfering with host-parasite interactions. This has provided insight into a dual process of natural selection and co-adaptation of polymorphisms occurring in the malaria parasite and its human host, to maintain genetic diversity. This review discusses recent findings on genetic modifiers shown to be significantly associated with and relevant to the diverse clinical outcomes of *P. falciparum *malaria. It is focused on the new gene polymorphisms found via genome-wide (GW) association studies (GWAS), case control studies on different populations and provides new perspectives for the different studies presented.

**Table 1:** Genetic mutations involved in susceptibility/resistance to *P.falciparum *malaria

Gene *(Symbol)*	Phenotype	Proposed protective mechanisms	References
Haemoglobin C *(HbC)*	↓UM & ↓SM	Reduced cyto-adherence of infected erythrocytes	[29,47]

Haemoglobin E *(HbE)*	↓SM, ↓parasitaemia	Reduced erythrocyte invasion by merozoites, lower intra-erythrocytic parasite growth, and enhanced phagocytosis of infected erythrocytes.	[48,49]

Haemoglobin S *(HbS)*	↓UM & ↓SM	Selective sickling of infected sickle trait erythrocytes leading to enhanced clearance by the spleen. Reduced erythrocyte invasion, early phagocytosis, and inhibited parasite growth under oxygen stress in venous micro vessels. Enhancement of innate and acquired immunity.	[7,50]

α-thalassaemia *(α-thal)*	↓SM & ↓SMA	Reduced resetting. Increased micro-erythrocyte count in homozygotes reduces the amount of haemoglobin lost for given parasite density, thus protecting against severe anaemia.	[51-53]
		
β-thalassaemia *(β-thal)*	↓SM		[54,55]

Glucose-6-Phosphate dehydrogenase *(G6PD)*	↓UM & ↓SM	Increased vulnerability of the G6PD deficient erythrocyte to oxidant stress causes its protection against parasitization.	[56-59]

Pyruvate kinase *(PKLR)*	↓parasitaemia	Invasion defect of erythrocytes and preferential macrophage clearance of ring-stage-infected erythrocytes.	[60]

Ovalocytosis *(SLC4A1)*	↓SM & ↓CM	Inhibition of merozoite entry into the red cell, impairment of intracellular parasite growth and prevention of the erythrocyte lysis that occurs with parasite maturation, leading to release of merozoites into the blood stream.	[61,62]
		
Elliptocytosis	↓SM		[63]
		
Glycophorins A *(GYP ABC)*	↓SM		[64,65]

Blood Groups *(ABO)*	↓SM	Reduced *P. falciparum *rosetting.	[66-68]
Haptoglobin *(HP)*	↓SM	Oxidative damage to uninfected cells might be more marked in HP polymorphic individuals since HP proteins bind less efficiently to Hb, increasing premature destruction of erythrocytes and stimulating cytokine release by these circulating cells.	[69-71]

Nitric oxide synthase 2 *(NOS2)*	↓SM	Increased NO production induces Th1 cytokines which activate macrophages and could thus be an anti-malarial resistance mechanism.	[72,73]

haem oxygenase I *(HO-1)*	↓CM	Release of free haem in the blood stream.	[13,14]

### Gene mutations involved in susceptibility and resistance to *P. falciparum *malaria

It has been shown that severity of several malaria infections (such as asymptomatic, CM and SMA) varies significantly between individuals and between populations [5]. Several gene mutations causing inherited diseases or traits have been reported to influence malaria severity (Table 1) [6]. Mutations in these genes have been linked to erythrocytes including haemoglobin (Hb) variants, or related to proteins such as haptoglobin and Nitric Oxide metabolism. For example, the heterozygote HbAS (sickle cell trait) which protects against severe malaria (SM) [7-10] is widespread in malaria endemic regions as a result of natural selection over generations [11]. It has also been shown that the rate limiting enzyme haem oxygenase I (HO-1), responsible for the catabolism of free haem in the body, plays an important moderator role in malaria and is also important in the pathophysiology of haemolytic diseases, such as sickle cell disease [12-15]. Epistatic interactions between genetic disorders of haemoglobin (HbAS, thalassaemia, HbE, etc.) show evidence of heterozygote protection from malaria (Table 1) [16] and protection against malaria by the sickle cell trait is removed if there is co-inheritance of alpha-thalassaemia [17]. These studies emphasize the underlying complexity of the field and therefore stress the need for newer methods of genomic analysis. Although malaria resistance gene mutations have been well studied, genes associated with red cell disease severity deserve further scrutiny.

### Genome-wide linkage and association studies in malaria

Some landmark genome wide linkage (GWL) and association studies (GWAS) have been conducted in recent years in African, European, Asian European and Asian populations. Application of GWAS to populations in Africa could provide insights into pathways controlling resistance to malaria as well as genetic origins of related diseases.

A GW gene expression study conducted by Griffiths *et al *[18] showed that a cluster of genes were expressed in correlation with absolute neutrophil count. The neutrophil-related gene region contained genes predicted to encode mediators of innate and adaptive immunity, including those for cytokine receptors (IL1R2, IL18R1, and IL6R), Toll-like receptors (TLR1 and TLR4), heat-shock proteins (HSPA1A and HSPA1L), the acute-phase proteins ferritin (FTL) and alkaline phoshatase (ALPL), and intracellular signaling factors (NFKBIA, JUNB, and FOSL2). The region also contained genes linked with neutrophil activation, such as those for grancalcin (GCA), a degranulation marker (CD66), and MAPK14 kinase. Many other genes, whose transcript levels were previously noted in human leukocyte models of in vitro bacterial infection, were also present (e.g., those for adrenomedullin, pre-B cell colony enhancing factor, and tumor necrosis factor-associated inducible protein) [18]. Based on changes in expression patterns of these genes, febrile and convalescent children could be assigned to distinct groups, indicating that neutrophil response plays a role in acute malarial infection. A second gene cluster was found to be associated with parasite density such that children with malaria could be distinguished from non-malaria patients, on the basis of different expression profiles. The cluster included genes encoding for pro-inflammatory molecules, markers of cellular stress and pro-apoptotic mediators [18]. Results also identified host gene responses (*HMOX1, HSPCB*, and *TNFRSF6*) that were related to the level of plasmodium parasitaemia. These studies identified several interesting candidate genes for further association studies to determine their roles in malarial immunity and pathogenesis.

GW linkage analyses of malaria infection severity revealed significant linkages to chromosome 10p15.3-14 and chromosome 13q [19]. Despite previous convincing results, no evidence of linkage was obtained for the 5q31 region to parasite density. Interestingly, a weak signal of linkage was observed for this region to malarial anaemia. The authors emphasized the difficulty of accurately defining the phenotype of malaria infection that could partially explain the divergence of linkage results [20].

Another GW linkage screening was carried out in a longitudinal survey of parasitological and clinical data from two independent Senegalese villages, Dielmo and Ndiop, that differ in ethnicity, malaria transmission and endemicity [21]. Analysis of several malaria-related phenotypes both during clinical disease and asymptomatic infection showed evidence of strong genetic contribution to both phenotypes studied. Asymptomatic parasite density showed linkage to chromosome 5q31, confirming previous findings [20]. Suggestive linkage values were also obtained: episodes of clinical malaria disease were linked to chromosome 5p15 and 13q13, while the maximum parasite density during asymptomatic infection was linked to chromosome 12q21. While regions of linkage showed little overlap with genes known to be involved in SM, the four regions appeared to overlap with regions linked to asthma or atopy related traits, suggesting that common immune related pathways may be involved. These newly identified linkage regions are interesting, but will require validation by independent studies. Also, fine mapped association studies are required to identify the genes underlying these linkages [21]. Ockenhouse *et al *investigated aspects of the earliest responses to malaria infection at the molecular level, and suggested an important role of innate and adaptive immune responses in different stages of infection [22].

Several inter-ethnic comparative studies showed that the Fulani population from West Africa is more resistant to *P. falciparum *malaria than are other sympatric ethnic groups [23]. The analysis of the immune response to *P. falciparum *sporozoite and blood stage antigens, as well as non-malaria antigens, revealed higher immune reactivity in the Fulani and that higher resistance to malaria among them could derive from a functional deficit of T-regulatory cells [23]. In this study, the results suggest that T-regulatory cell activity could be central in the control of malaria infection also in populations exposed to naturally high *P. falciparum *transmission. Furthermore, this study highlights the existence of clear-cut differences in strategic pathways of the immunoregulatory network between sympatric populations differing in their genetic background and degree of susceptibility to malaria. A higher resistance against *P. falciparum *malaria could have been the driving selective force of this disorder.

Jallow *et al *conducted a GWAS of SM in 2,500 children from The Gambia, which was replicated in an additional 3,400 children [24]. Besides the considerable population stratification found, their result show that signals of association at known malaria resistance loci were greatly attenuated due to weak linkage disequilibrium (LD). Conversely, the GW association analysis did not identify any of the well-known erythrocyte variants that have been selected by malaria, other than HbS. They explained this partly by population genetic factors; for example, the Duffy FY*O allele has reached fixation in The Gambia, whereas other variants, such as those affecting haemoglobin C and Southeast Asian ovalocytosis, are rare or absent in this population. No associations were found at *G6PD *and *HBA1-HBA2*, the loci for glucose-6-phosphate deficiency and β^+^-thalassaemia, respectively, possibly due to the lack of fine mapping of the SNPs (single nucleotide polymorphisms) dataset within these regions. The group genotyped the SNP rs1050828, a *G6PD *coding polymorphism, that was suggested to be a marker for protection against SM [25]. The minor allele frequency of rs1050828 in the Gambian control sample was 0.03, considerably lower than for samples from Kenya (0.18) and Malawi (0.19) [24]. The power to detect association with rs1050828 in The Gambia was affected by this low allele frequency, and the results were consistent with a modest protective effect although not statistically significant.

The rs8176719 genotype (a splice-site insertion in the *ABO *gene) is consistent with previous studies, which found that individuals of different populations who are not of blood group O, have 1.2-fold increased risk of SM [26]. Other SNP associations (on *CD36, CD40LG, CR1, ICAM1, IL22, NOS2*, and *TNF*) have been reported for malaria, but have not been conclusively replicated in large studies across different populations, and are mostly thought to be markers rather than true causal variants. The authors attribute this in part to low tagging efficiency of the Affymetrix 500 K array used and low statistical power, particularly low allele frequencies. In addition, they identified several significant association regions other than *HBB *(Hb-beta locus): on chromosome 2q37 (with the closest genes *SPATA3, LOC257407, PSMD1 *and *GPR55*), on chromosome 5p12 (in a region that has a number of genes encoding proteins of unknown function) and on chromosome 14q21 (in an area with few genes) [26]. Further investigations are needed to prove that polymorphisms in these genes significantly affect malaria outcomes.

### Gene polymorphisms associated with protection against *P. falciparum *malaria

Recently published polymorphisms that are significantly associated with susceptibility and resistance to *P. falciparum *malaria are summarized in additional file 1. The large majority of the polymorphisms found in these reports were mainly genes directly or indirectly involved with host immunity, including human leukocyte antigen system (HLA) genes, cytokine genes, complement regulatory genes and endothelial receptor genes. These polymorphisms do not cause host genetic pathology themselves, but are associated with malaria severity. A description of the function of their corresponding gene products is summarized in Additional file 2.

Although malaria remains a devastating disease responsible for high global mortalities, only few association studies have been reported on malaria phenotypes and polymorphisms of candidate loci. It is well established that in some cases as in haemoglobinopathies, despite the lack of a consensus on the mechanism of protection, the actual protective role has been identified and solid epidemiological evidence has been reported (Table 1). However, much information remains to be obtained for many genes related to the red cell surface, oxidative stress, cyto-adherence and immune response associated with malaria. In fact, only a few of the associations reviewed in Additional file 1 have been tested in independent studies in different populations and even when replication has been attempted, results have often been conflicting; either the initial finding could not be replicated or a polymorphism initially associated with increased risk of SM in a study was associated with protection against SM in another study. Finally, in some instances the genotypic and/or haplotypic patterns of association varied across different studies/populations (Additional file 1). These inconsistencies can be explained in several ways:

### Sample size and source

It has been established that GW screening conducted on large sample sizes and in multiple populations have greater potential to be more informative. Sometimes, the lack of association can be a false negative result due to lack of statistical power. Another difficulty is that in most cases, when population based phenotype-genotype relationships are studied, it is assumed that the population is genetically isolated. The extensive genomic diversity within Africa and across different continents complicates the situation. Association signals for a genotyped variant could show different patterns in different populations, due to local variation in haplotype structure and linkage disequilibrium architecture. Furthermore, it is important to emphasize that the genetic basis of susceptibility/resistance to malaria is due to a broad range of susceptibility/protective genes, each resulting in small population effects, which may be missed at low analytical power, such as low allele frequencies. In both cases, the use of larger sample sizes would certainly be of great value.

### Population substructure and/or admixture

Inconsistency of results may also be due to issues of population structure and/or admixture [27]. This is particularly true in African populations, where genetic diversity is exceptionally high. Studies on the same population but in different areas (endemic versus non-endemic regions) have revealed differences in host response to *P. falciparum *[28]. There is also the possible impact of variation in environment (climate, nutritional status) which results in variation of pathogen epidemiology and which becomes more relevant in the rapidly changing socio-economic forces impacting these populations. Furthermore, the lesson learned about genetics of haemoglobinopathies illustrates how distinct malaria resistant alleles have emerged in different populations due to selective pressure, with HbS being found much more endemic in Africa (on four distinct haplotypes) than in Asia, and the opposite for HbE, or again with the relative prevalence of HbS and HbC varying greatly between neighboring countries and even villages [29]. Fine mapping of GW SNP studies in several different populations, and re-confirmed sequencing of regions of special interest, could provide accurate representations of the genetic background and, therefore, a more effective interpretation of association results.

### Variation in sampling methodology

Another very important cause of discrepancy is the way severity of malaria is defined. Each of the reported studies classifies malaria severity on the basis of different criteria and thus a common unified and systematic international classification is absolutely needed. This must be considered when studying a large number of individuals across geographical populations. Factors like age may have an important effect on results. Studies that have both children and adults will yield very different results from those involving only adults. The setting where samples are collected may also impact results (urban vs. rural, hospital/clinic based vs. population random cluster sampling).

The establishment of a well-characterized tissue repository with accompanying databases and a robust data-sharing plan would be of great benefit for standardizing phenotype definition, genotyping technology, experimental and analytical plans across multiple sites, to improve the power of the GW studies and ensure reproducibility of the results.

A major driving force in this field of research has been the recent availability of the genome variations data and other information free on line. Databases like *dbSNP *and *Nucleotide, Genome *and *Entrez *and *PubMed *among others have helped scientists around the world, especially in Africa, to obtain uniform data on host genetics and infectious diseases. Another factor is the benefit of using high throughput technologies and automated microarrays that can screen whole genomes simultaneously. This kind of costly research will only be possible if serious, consistent and strong collaborative effort is encouraged. Infrastructure to conduct case-controlled GW and multi-centre association studies on malaria susceptibility and resistance must be established where the disease in endemic. Such important studies are very necessary and will provide new insights into the effects of genetic variation on malaria susceptibility and on molecular mechanisms for protective immune responses [30]. However, some studies using these technologies missed some of the significant associations unequivocally determined by classical genetics [24] and the role of regions of the genome involved in malaria resistance, such as alpha-thalassaemia or G6PD deficiency was missed. This raises doubts about the sensitivity of the approach employed. The major limiting factor, at all stages of GW association analysis in Africa, is the need for population-specific data on genome sequence variation. In the near future, this limiting factor should be overcome by advances in genome sequencing technologies, through initiatives such as the 1000 Genomes Project.

### Sensitivity of methods

Until GW studies picked up sickle cell trait as a bench mark reference protective factor, utility of these studies in understanding genes associated with malaria severity continues to be limited to identifying only broad associations within the genome. It will be beneficial to use sickle cell trait to assess the power of GWAS. There is also a need for associating gene polymorphism to expressed protein variants using sensitive immunoassay procedures that could identify clusters of biomarker proteins associated with susceptibility and severity of malaria. This approach could be used to establish panels for predicting potentially fatal malaria. Multiplex immunoassay procedures and proteomic technologies should be combined with GWAS and new diagnostics for detecting susceptibility to fatal disease [31]. For example, recent human and murine gene knock out studies suggest that plasma levels of Interferon inducible protein 10 (IP-10; CXCL10) [32], soluble TNF receptor 2 (sTNF-R2) [33] and soluble Fas (sFas) [34] predict risk of malaria related mortality and may be potential biomarkers of CM severity. Additionally, angiogenic factors such as vascular endothelial growth factor (VEGF) were found to be protective against CM associated mortality and may be considered for adjunctive therapy, to improve treatment outcomes in CM patients [35]. Other potential biomarker candidates are interleukin-10 (IL10) and the Granulocyte colony-stimulating factor (G-CSF), cytokines which are associated with susceptibility to asymptomatic malaria during pregnancy [36]. Another recent study has suggested a prominent role for CXCL4 and CXCL10 in the pathogenesis of fatal CM [37]. Clearly, assessment of polymorphisms associated with these significant risk factors or prognostic biomarkers could predict fatal disease outcomes and must be investigated in malaria endemic population. Recent studies have implicated several other genes in the pathogenesis of SMA, CM and placental malarial. It is very important to determine if any gene polymorphisms are associated with these candidate genes.

The Macrophage migration inhibitory factor (MIF) has also been suggested to have a protective role in pathogenesis of malaria [38]. MIF is a multifunctional cytokine which is an important regulator of immune and inflammatory responses in a number of human diseases, such as sepsis, rheumatoid arthritis, cancer and inflammatory neurological diseases [39]. The potential role of MIF in the pathogenesis of malaria anaemia became apparent in an experimental study using a mouse model in which high MIF levels were associated with malaria anaemia [40]. Human studies conducted on African children reported lower levels of MIF in malaria infected children compared with healthy asymptomatic children [41]. Another study demonstrated a decline in MIF levels during experimental malaria infection using healthy European volunteers [42]. The role of circulating MIF, gene polymorphisms as well as potential interactions with other factors in the pathogenesis of CM and its outcome, need to be further investigated.

### Role of co-infection in hosts

Several gene pathways reported here are also involved in host responses to bacterial infection. However, caution must be exercised when defining severity phenotype. For example, in high transmission areas most children will have asymptomatic carriage of parasites in their blood such that any acute illness may be ascribed to malaria. It is becoming clear that many of these children carry bacterial sepsis along with the malaria [43]. It will, therefore, be necessary to check for bacterial infection to exclude combined effects during recruitment of volunteer participants in case control studies. Another important factor is to determine whether individual patients have multiple *Plasmodium *infections since each species may present different etiologies. For example, it would be very interesting to determine host factors mediating susceptibility to *Plasmodium vivax *malaria, which is fast becoming recognized as a major cause of SM in Southeast Asia and elsewhere [44,45].

### Gene polymorphisms associated with multiple diseases

A recent review of gene polymorphisms involved in different phenotypes of sickle cell disease [46] revealed that many genes or pathways mediating sickle cell disease severity are also involved in malaria severity/resistance. For example, the *TNFα *(-308A, rs1800629) polymorphism, which reduces SM, CM, SMA and iron deficiency anaemia, is also protective against large vessel stroke in sickle cell disease. Findings such as these, which reveal genetic similarities across related diseases, will be valuable for identifying important diagnostic biomarkers and for population comparisons.

## Conclusion

It is clear that genetically-based alterations conferring protection against malaria have led to co-adaptation of various human populations with widespread malaria parasites. This co-adaptative process has resulted in benefits for host (protection) and parasite (reduced virulence/chronicity). A global collaborative effort or consortium must be made to collect information about involvements of biomarkers in malaria susceptibility. This collaboration should include phenotype and clinical data as well as genomics, proteomics, metabolomics and parasitomics. GW research on protective polymorphisms against malaria will lead to better understanding of the mechanisms underlying malaria severity, which can be used in developing novel therapeutic solutions.

## List of abbreviations

SMA: severe malaria anaemia; CM: cerebral malaria; Hb: Haemoglobin; HbAS: Haemoglobin AS or sickle cell trait; G6PD: glucose-6-phosphate dehydrogenase; GW: genome-wide; SM: severe malaria; HLA: human leukocyte antigen system; GWL: genome wide linkage; GWAS: genome wide association studies; SNP: Single Nucleotide Polymorphism; LD: linkage disequilibrium

## Competing interests

The authors declare that they have no competing interests.

## Authors' contributions

AD planned the review, assessed recent studies using different publications research tools, collected and analyzed the data and drafted the manuscript. JMH participated in coordination of the review and helped in the critical views of the discussion. NOW, SAI and TVA participated in the design and coordination of the review and corrections and suggestions. JKS participated in the planning, design, coordination, supervision, interpretation of data and revised the manuscript for important intellectual content. All authors helped to draft and correct the manuscript as well as read and approved the final manuscript.

## Supplementary Material

Additional file 1 **Review of gene polymorphisms reported to date to be significantly associated the host phenotype of susceptibility/resistance to *P. falciparum *malaria**. ↑increase, ↓decrease, SM: Severe Malaria. SMA: Severe Malaria Anaemia. CM: Cerebral Malaria. MM: Mild Malaria. UM: Uncomplicated Malaria [74-121]. Click here for file

Additional file 2 **Summary of gene functions **[122-137]. Click here for file
