# Supplementary material for: Genetic polymorphisms linked to susceptibility to malaria
Source: Malar J. 2011 Sep 19;10:271. doi: 10.1186/1475-2875-10-271 (PMC3184115; doi:10.1186/1475-2875-10-271)
Supplement: Additional file 1 — Review of gene polymorphisms reported to date to be significantly associated the host phenotype of susceptibility/resistance to P. falciparum malaria. ↑increase, ↓decrease, SM: Severe Malaria. SMA: Severe Malaria Anaemia. CM: Cerebral Malaria. MM: Mild Malaria. UM: Uncomplicated Malaria [74-121]. [file 1475-2875-10-271-S1.PDF]

Additional file 1. Review of gene polymorphisms reported to be significantly associated the host phenotype of susceptibility/resistance to *P. falciparum* malaria. ↑increase, ↓decrease, SM: Severe Malaria. SMA: Severe Malaria Anaemia. CM: Cerebral Malaria. MM: Mild Malaria. UM: Uncomplicated Malaria.

Function	Gene	Chromosome (GeneID)	SNP or allele	*p* value	Phenotype	Population	Sample Size	Ref
Immune system	HLA-B	6p21 (3106)	B*53	0.03	↓SM	The Gambia	2035	[74]
			B*35	<0.001	development of specific immunity	The Gambia	795	[75, 76]
						Mali	115	
	HLA-DRB1	6p31.3 (3123)	*1302	0.0023	↓SM	The Gambia	2035	[74]
	TNFa	6p21.3 (7124)	rs361525 -238A	<0.001	↓SM & ↓CM ↓SMA	The Gambia	420	[77]
			rs1800629 -308A	0.001			376	[78]
				0.01	↓iron deficiency anaemia		780	[79]
			rs1799964 T-1031C	0.013	↑SM	India	361	[28]
			rs1800630 C-863A	0.003			361	
	IFNG	12q14 (3458)	−183T	0.009	↓CM ↓parasite infection	Mali	136	[80]
			−183G/T	0.013			136	
			(CA)14	0.073			136	
	IFNGR1	6q23.3 (3459)	−56	0.006	↓CM	The Gambia	1131	[81]
	IRF1	5q31.1 (3659)	rs10065633 rs2706384 rs10213701	0.02	↓UM and SM: control of parasite infection	Fulani vs Mossi Burkina Faso	780	[82]
				0.02	not associated with SM	The Gambia	555	[83]
				0.06		Kenya	204	
				?		Malawi	202	
	CD40LG	Xq26 (959)	−726C	0.002	↑SM & ↑CM ↑SMA	The Gambia	957	[84]
	IL1A	2q14 (3552)	+4845G>T	0.035	↑SM & ↑CM ↑SMA		376	[85]

IL1B	2q14 (3553)	+3953C>T	0.03	↑SM & ↑CM ↑SMA	Ghana	461	[86]
			0.01	↑parasitaemia			
			-	not detected	Thailand	312	[87]
		-31C>T	0.82	not associated			
IL1RA	2q12 (3554)	VNTR	0.88				
IL4	5q31.1 (3565)	−524T	<0.001	Difference between tribes	Fulani vs Mossi & Rimaibe – Burkina Faso	159	[88]
		-589C/T	0.018	↑total IgG, ↑total IgE	Mossi - Burkina Faso	159, 580	[88, 89]
		-590 T	<0.0001	Difference between tribes	Fulani vs Dongo – Mali	426	[90]
			0.046	↑parasitaemia	Thailand	279	[91]
		+33T/-590T	0·03	↑total IgE in CM	Ghana	476	[92]
IL10	1q31-q32 (3586)	-1082A/G -819T/C -592A/C	G/C/C 0.042	↓SMA	Kenya	375	[93]
IL22	12q15 (50616)	+708T	0.016	↓SM	The Gambia	676	[94]
		-1394G	0.05	↑CM			
FCGR2A / CD32A	1q23 (2212)	131R/H	0.001	↑ anti-malarial IgG2, IgG3, IgG1	Fulani - Sudan	351	[95]
		131R	0.0021	↓parasitaemia	Kenya	182	[96]
		131R/R	0.04	↑SM, ↑CM	Sudan	256	[97]
		131H/H	0.01	↓SM MM			
			0.03	↑SM	The Gambia	1431	[98]
		131H/H / NA2	0.012	↑CM	Thailand	466	[99]
		131H	0.01	↓SM	India	361	[28]
FCGR2B / CD32B	1q23 (2213)	T/T232	0.0001	↓parasitaemia, ↓SM	Kenya	473	[100]
TLR4	9q32-q33 (7099)	rs4986790 G299	0.05	↓SM	Ghana	580	[101]
		rs4986791	0.02				

			I399					
	TLR1	4p14 (7096)	(T1805G) I602S	0.003	↑MM	Brazil	304	[102]
	TLR6	4p14 (10333)	rs5743810 S249P	0.01	↑MM			
	TLR9	3p21.3 (54106)	rs1870884 -1486C	<0.001	↑parasitaemia			
	TIRAP	11q24.2 (114609)	rs8177374 S180L	0.49	not associated	Ghana	1095	[103]
				0.19		Germany	1114	
				0.22		Bangladesh		
				0.11		Turkey		
	CYBB	Xp21.1 (1536)	TA11	0.07	↑SM & ↑SMA	Gabon	183	[104]
			TA16	0.04				
Complement Regulatory	MBL2	10q11.2-q21 (4153)	#54(2B) /#57(2C)	0.04	↑SM	Gabon	200	[105]
			-221 AA/OO	0.02	↑parasitaemia ↓blood glucose	Ghana	551	[106]
			#57(2)C	0.007	↑SM, ↑susceptibility on infection		870	[107]
	CR1	1q32 (1378)	CR1 deficiency	0.0001	↓rosetting	COS-7 cells	-	[108]
			L allele G3650	0.01	↓SM ↓SMA	Papua New Guinea	358	[109]
Endothelial receptors	ICAM1	19p13.3-p13.2 (3383)	rs5498 exon 6 (G allele)	0.016	↑SM	India	552	[110]
				0.097		Nigeria	223	[111]
			Rs5491 (Kilifi) (M29)	0.17	not associated	The Gambia	4058	[112]
						Malawi		
						Kenya		
				>0.5		Senegal	878	[113]
				0.012	protective	Gabon	200	[114]
				0.0042	↑CM	Kilifi, Kenya	547	[115]
	PECAM1/CD31	17q23 (5175)	Rs668 L125V	0.0001	↑SM	India	552	[110]
				0.02	↑CM vs SM	Thailand	475	[116]
				>0.5	not associated	Kenya	396	[117]

					Papua New Guinea	442	
		rs12953 S563N	0.01	↑CM vs SM	Thailand	475	[116]
CD36	7q11.2 (948)	rs1334512 (-53)	0.004	↑SM	India	552	[110]
			0.05	↓CM	Thailand	475	[118]
		rs2151916 (-14)	0.016	↓CM			
		in3(TG)$_{12}$	0.0069	↓CM			
		Exon 1a	0.00056	↑SM	India	552	[110]
		T1264G	0.04	↓CM	Kenya	514	[119]
					The Gambia	845	
		G1439C	0.01	↓CM			
		T188G	0.036	↓SM	Kenya	693	[120]
FUT9	6q16 (10690)	rs3811070	0.028	↓placental malaria infection	Mozambique	360	[121]
