# Supplementary material for: Genetic polymorphisms linked to susceptibility to malaria
Source: Malar J. 2011 Sep 19;10:271. doi: 10.1186/1475-2875-10-271 (PMC3184115; doi:10.1186/1475-2875-10-271)
Supplement: Additional file 2 — Summary of gene functions [122-137]. [file 1475-2875-10-271-S2.PDF]

Additional file 2: Summary of gene functions.

Gene product (Name)	Function
Human Leukocyte Antigen *(HLA)*	• HLA classes I and II molecules expressed on the surface of all nucleated cells and specialized antigen presenting cells.<br>• Responsible for recognition of foreign antigens through effective T cell response.<br>• All genes encoding for the class I (HLA-A, HLA-B and HLA-C which present peptides from inside the cell) and of class II molecules (HLA-DP, HLA-DQ, and HLA-DR which present antigens from outside of the cell to T-lymphocytes) are located on the human Major Histocompatibility Complex (MHC) on chromosome 6. These genes are known to be the most polymorphic genes in man.
Tumor Necrosis Factor-alpha *(TNFα)*	• A cytokine that plays an important role in the pathogenesis of SM [122] while fatal CM is associated with high circulating levels of this cytokine [122, 123].<br>• Different combinations of allelic variants may determine the severity of the clinical outcome of SM [77].<br>• TNF appears to be a risk factor for iron deficiency and iron deficiency anaemia in children in a malaria-endemic environment which may be due to a TNF-induced block in iron absorption [79].
tumor necrosis factor receptor superfamily, member 1B (TNFR2)	• Member of the TNF-receptor superfamily. TNFR2 protein and TNF-receptor 1 form a hetero-complex that mediates the recruitment of two anti-apoptotic proteins, c-IAP1 and c-IAP2, which possess E3 ubiquitin ligase activity.<br>• The function of IAPs in TNF-receptor signaling is unknown; however, c-IAP1 is thought to potentiate TNF-induced apoptosis by the ubiquitination and degradation of TNF-receptor-associated factor 2, which mediates anti-apoptotic signals.
Fas (TNF receptor superfamily, member 6) *(FAS)*	• Member of the TNF-receptor superfamily. This receptor contains a death domain. It has been shown to play a central role in the physiological regulation of programmed cell death, and has been implicated in the pathogenesis of various malignancies and diseases of the immune system.<br>• The interaction of this receptor with its ligand allows the formation of a death-inducing signaling complex that includes Fas-associated death domain protein (FADD), caspase 8, and caspase 10.<br>• The auto-proteolytic processing of the caspases in the complex triggers a downstream caspase cascade, and leads to apoptosis. This receptor has been also shown to activate NF-kappaB, MAPK3/ERK1, and MAPK8/JNK, and is found to be involved in transducing the proliferating signals in normal diploid fibroblast and T cells.
Interferon-γ *(IFN-γ)*	• Key cytokine mediator of anti-parasitic response effect [124]<br>• Malaria induced IFN-γ production seem to be associated with protection against re-infection and reduced risk of severity of the disease [125].
IFN-γ receptor *(IFNGR1)*	• α subunit of the IFN-γ receptor that plays an important role in ligand binding, receptor trafficking and

	signal transduction [126].
Transcription factor interferon regulatory factor 1 *(IRF1)*	• Functions as a transcription activator of genes induced by interferons alpha, beta, and gamma.[127]<br>• Has been shown to play roles in regulating apoptosis..
CXCL10 chemokine ligand 10 *(IP-10)*	• Chemokine of the CXC subfamily and ligand for the receptor CXCR3.<br>• A.K.A. IP-10 for interferon-g-inducible 10 kDa protein.<br>• Binding of CXCL10 protein to CXCR3 results in pleiotropic effects (stimulation of monocytes, natural killer and T-cell migration, and modulation of adhesion molecule expression).
CXCR3 chemokine (C-X-C motif) receptor 3 *(CXCR3)*	• G protein-coupled receptor with selectivity for CXCL10, Mig (monokine induced by interferon-g) and I-TAC (interferon-inducible T cell a-chemoattractant).<br>• CXCL10, Mig and I-TAC belong to subfamily of CXC chemokines.<br>• Binding of chemokines to this protein induces cellular responses involved in leukocyte traffic, integrin activation, cytoskeletal changes and chemotactic migration.<br>• CXCL10, Mig and I-TAC are produced by local cells in inflammatory lesion and participate in the recruitment of inflammatory cells.
CXCR4 chemokine (C-X-C motif) receptor 4 *(CXCR4)*	• CXC chemokine receptor specific for stromal cell-derived factor-1.<br>• Mutations in CXCR4 associated with WHIM (warts, hypogamma globulinaemia, infections, and myelokathexis) syndrome.
Vascular Endothelial Growth Factor A *(VEGFA)*	• PDGF/VEGF growth factor family members encode a protein often found as a disulfide linked homodimer.<br>• Glycosylated mitogen protein that specifically acts on endothelial cells and has various effects, including mediating increased vascular permeability, inducing angiogenesis, vasculogenesis and endothelial cell growth, promoting cell migration, and inhibiting apoptosis.
CD40 ligand *(CD40LG)*	• Glycoprotein involved in B cell proliferation, antigen presenting cell activation, and immunoglobulin isotype switching.<br>• This locus showed signatures of low genetic diversity within African populations [128].
Interleukin-1 (IL1-α and IL1-β)	• Important regulator of the innate immunity.<br>• Encoded by two genes (IL1A and IL1B) located on chromosome 2q14 in a cluster containing genes encoding for IL1 receptors (IL1R1 and IL1R2), and the IL1 receptor antagonist (IL1RN) among other homologues of this gene family [129].
Interleukin-4 *(IL4)*	• Cytokine that induces Ig class switching from IgM/IgG to IgE and enhances the antigen-presenting capacity of B lymphocytes.<br>• The gene is previously shown to be linked to *P. falciparum* infection [20] and to elevated total IgE levels [130].
Interleukin-10 *(IL10)*	• IL10 promoter haplotypes may mediate susceptibility to SMA and may cause functional changes in circulating IL10, TNFα and IL12 levels in children with *P. falciparum* malaria [93].

Interleukin-22 (IL22)	• IL22 is a cytokine of the IL10 family, produced by Th1 cells. Its properties include activation of STAT1 and STAT3 and upregulation of acute phase reactants. It has been thought to act mainly at the level of peripheral innate immunity, but it is possible that in malaria, it plays a different role. [131, 132]
Fcγ receptors	• Binds IgG and may be activatory (FcγRI, IIa, IIIa, IIIb, and IV) or inhibitory (FcγRIIb).<br>• FcγRIIb inhibits many features of the immune response, including FcγR-mediated phagocytosis, proinflammatory cytokine production, antigen presentation, and antibody responses.<br>• Mice deficient in FcγRIIb demonstrate hyperactive immune responses and are prone to autoimmune disease, particularly for systematic lupus erythematosus (SLE) [133], but show increased resistance to bacterial infections and parasite clearance [100].
Toll-like receptors (TLRs)	• Key host molecules involved in the induction of innate immune responses to microbial ligands [134].<br>• Upon recognition of molecular motifs specific for microbial molecules, TLR mediate pro-inflammatory cytokine secretion and enhance antigen presentation[135].<br>• *P. falciparum* glycosylphosphatidylinositol was reported to induce signaling via both TLR2 and -4 and haemozoin-induced immune activation was reported to involve TLR9 [136].
TLR downstream signal transducer (*TIRAP)*	• A SNP (S180L) has been reported to be common in Europeans and Africans and linked to the risks of heterogeneous infectious diseases including malaria, tuberculosis, bacteraemia, and invasive pneumoccocal disease in heterozygous mutation carriers.
Cytochrome beta b-245 *(CYBB)*	• Reactive oxygen intermediates play a major role in nonspecific innate immune response to invading microorganisms such as *P. falciparum*. [104].
mannose-binding protein *(MBL2)*	• MBL deficiency is the most common congenital immunodeficiency in humans and has been shown to predispose them to infections, particularly in children immuno-compromised.
Complement receptor 1 *(CR1)*	• Immune-regulatory protein present on erythrocytes and a variety of leucocytes involved in complement activation and clearance of immune complexes. CR1 plays an important role in protecting host cells from attack by complement.
Intercellular adhesion molecule-1 *(ICAM1)*	• Cell Adhesion Molecules (CAMs) are proteins located on the endothelial cell surface and are involved in cell adhesion.<br>• ICAM1 is the main receptor for infected erythrocytes on the endothelium.<br>• In vital organs, the binding of parasitized cells to the endothelium via ICAM1 may lead to severe disease and death.
platelet-endothelial cell adhesion molecule *(PECAM1 or CD31)*	• Binding receptor for *P. falciparum* to the endothelium [137].
CD36	• Mutations in this gene might have a complex effect on malaria infection by decreasing parasite sequestration, and by decreasing host immune responses. [120].

Fucosyl-transferase 9 *(FUT9):*	• Codes for a fucosyl-transferase that catalyze the last step in the biosynthesis of the Lewis-x antigen, which forms part of the Lewis blood group-related antigens.
haemoglobin alpha *(HBA)*	• The alpha-2 (HBA2) and alpha-1 (HBA1) coding sequences are identical. These genes differ slightly over the 5' untranslated regions and the introns, but they differ significantly over the 3' untranslated regions.<br>• Two alpha chains plus two beta chains constitute HbA, which in normal adult life comprises about 97% of the total haemoglobin;<br>• Alpha chains combine with delta chains to constitute HbA-2, which with HbF (foetal haemoglobin) makes up the remaining 3% of adult haemoglobin.<br>• Alpha thalassaemias result from deletions of each of the alpha genes as well as deletions of both HBA2 and HBA1; some non-deletion alpha thalassaemias have also been reported.
Haemoglobin beta *(HBB)*	• The normal adult haemoglobin tetramer consists of two alpha chains and two beta chains.<br>• Mutant beta globin causes sickle cell anaemia (HbS or HbC or HbE). Absence of beta chain causes beta-zero-thalassaemia. Reduced amounts of detectable beta globin causes beta-plus-thalassaemia.
Glucose-6-Phosphate dehydrogenase *(H6PDA-)*	• Cytosolic enzyme encoded by a housekeeping X-linked gene whose main function is to produce NADPH, a key electron donor in the defense against oxidizing agents and in reductive biosynthetic reactions.<br>• G6PD is remarkable for its genetic diversity. Many variants of G6PD, mostly produced from missense mutations, have been described with wide ranging levels of enzyme activity and associated clinical symptoms.<br>• G6PD deficiency may cause neonatal jaundice, acute haemolysis, or severe chronic non-sphaerocytic haemolytic anaemia.
Pyruvate kinase *(PKLR)*	• Catalyzes the trans-phosphorylation of phosphoenolpyruvate into pyruvate and ATP, which is the rate-limiting step of glycolysis.<br>• Defects in this enzyme, due to gene mutations or genetic variations, are the common cause of chronic hereditary nonsphaerocytic haemolytic anaemia.
Solute Carrier family 4 anion exchanger 1 *(SLC4A1)*	• Expressed in the erythrocyte plasma membrane, where it functions as a chloride/bicarbonate exchanger involved in carbon dioxide transport from tissues to lungs.<br>• The N-terminal domain is located in the cytoplasm and acts as an attachment site for the red cell skeleton by binding ankyrin. The glycosylated C-terminal membrane-associated domain carries out the stilbene disulphonate-sensitive exchange transport of anions. The cytoplasmic tail at the extreme C-terminus of the membrane domain binds carbonic anhydrase II.<br>• Many mutations in this gene are known in human, and these mutations can lead to two types of disease: destabilization of red cell membrane leading to hereditary sphaerocytosis, and defective kidney acid secretion leading to distal renal tubular acidosis. Other mutations that do not give rise to disease result in novel blood group antigens, which form the Diego blood group system. Southeast Asian ovalocytosis (SAO,

	Melanesian ovalocytosis) results from the heterozygous presence of a deletion in the encoded protein and is common in areas where *Plasmodium falciparum* malaria is endemic. One null mutation in this gene is known, resulting in very severe anaemia and nephrocalcinosis.
Blood Groups *(ABO)*	• Proteins related to the ABO blood group system. Which allele is present in an individual determines the blood group.<br>• The 'O' blood group is caused by a deletion of guanine-258 near the N-terminus of the protein, which results in a frameshift and translation of an almost entirely different protein.<br>• Individuals with the A, B, and AB alleles express glycosyltransferase activities that convert the H antigen into the A or B antigen. Other minor alleles have been found for this gene.
Glycophorins A (*GYP ABC*)	• Glycophorins A (GYPA) and B (GYPB) are major sialoglycoproteins of the human erythrocyte membrane which bear the antigenic determinants for the MN and Ss blood groups.<br>• In addition to the M or N and S or s antigens that commonly occur in all populations, about 40 related variant phenotypes have been identified.<br>• These variants include all the variants of the Miltenberger complex and several isoforms of Sta, as well as Dantu, Sat, He, Mg, and deletion variants Ena, S-s-U- and Mk. Most of the variants are the result of gene recombinations between GYPA and GYPB.
Erythrocyte Membrane Protein Band 4.1 *(EPB41)*	• The protein of this gene together with spectrin and actin, constitute the red cell membrane cytoskeletal network, playing a critical role in erythrocyte shape and deformability.<br>• Mutations in this gene are associated with type 1 elliptocytosis (EL1). Alternatively spliced transcript variants encoding different isoforms have been described for this gene.
Haptoglobin *(HP)*	• The gene encodes a preproprotein, which is processed to yield both alpha and beta chains, which subsequently combine as a tetramer to produce haptoglobin.<br>• Haptoglobin functions to bind free plasma haemoglobin, which allows degradative enzymes to gain access to the hemoglobin, while at the same time preventing loss of iron through the kidneys and protecting the kidneys from damage by hemoglobin.<br>• Mutations in this gene and/or its regulatory regions cause ahaptoglobinaemia or hypohaptoglobinaemia. This gene has also been linked to diabetic nephropathy, the incidence of coronary artery disease in type 1 diabetes, Crohn's disease, inflammatory disease behaviour, primary sclerosing cholangitis, susceptibility to idiopathic Parkinson's disease, and a reduced incidence of *Plasmodium falciparum* malaria.<br>• A similar duplicated gene is located next to this gene on chromosome 16. Multiple transcript variants encoding different isoforms have been found for this gene.
Nitric oxide synthase 2 *(NOS2)*	• Nitric oxide is a reactive free radical which acts as a biologic mediator in several processes, including neurotransmission and antimicrobial and antitumoral activities. This gene encodes a nitric oxide synthase which is expressed in liver and is inducible by a combination of lipopolysaccharide and certain cytokines.

haem oxygenase I *(HO-1)*	• An essential enzyme in haem catabolism, cleaves haem to form biliverdin, which is subsequently converted to bilirubin by biliverdin reductase, and carbon monoxide, a putative neurotransmitter.<br>• Haem oxygenase activity is induced by its substrate haem and by various non-haem substances.
Granulocyte colony-stimulating factor *(G-CSF)*	• Cytokine that controls the production, differentiation, and function of granulocytes.<br>• G-CSF also stimulates the production of white blood cells (WBC).
